# Aryl hydrocarbon receptor agonist indigo protects against obesity-related insulin resistance through modulation of intestinal and metabolic tissue immunity

**DOI:** 10.1038/s41366-019-0340-1

**Published:** 2019-04-03

**Authors:** Yi-Hsuan Lin, Helen Luck, Saad Khan, Pierre H. H. Schneeberger, Sue Tsai, Xavier Clemente-Casares, Helena Lei, Yann-Lii Leu, Yi Tao Chan, Hsing-Yu Chen, Sien-Hung Yang, Bryan Coburn, Shawn Winer, Daniel A. Winer

**Affiliations:** 1 Division of Chinese Internal Medicine, Center for Traditional Chinese Medicine, Chang Gung Memorial Hospital, Taoyuan, 33378 Taiwan; 2 School of Traditional Chinese Medicine, College of Medicine, Chang Gung University, Taoyuan, 33302 Taiwan; 3 Graduate Institute of Clinical Medical Sciences, College of Medicine, Chang Gung University, Taoyuan, 33302 Taiwan; 4 Division of Cellular & Molecular Biology, Diabetes Research Group, Toronto General Research Institute (TGRI), University Health Network, 101 College Street, Toronto, ON M5G 1L7 Canada; 5 Department of Immunology, University of Toronto, 1 King’s College Circle, Toronto, ON M5S 3B3 Canada; 6 Department of Medicine, Division of Infectious Diseases, University Health Network, Toronto, Canada; 7 Department of Laboratory Medicine and Pathobiology, University of Toronto, Toronto, ON M5S 1A8 Canada; 8 Graduate Institute of Natural Products, College of Medicine, Chang Gung University, Taoyuan, 33302 Taiwan; 9 Chang Gung Immunology Consortium, Chang Gung Memorial Hospital and Chang Gung University, Gueishan, Taoyuan, 33302 Taiwan; 10 Department of Laboratory Medicine, St. Michael’s Hospital, Toronto, ON M5B 1W8 Canada; 11 Department of Pathology, University Health Network, 200 Elizabeth Street, Toronto, ON M5G 2C4 Canada; 12 Buck Institute for Research on Aging, 8001 Redwood Boulevard, Novato, CA 94945 USA; 13 Present Address: 10-352 Toronto Medical Discovery Tower, 101 College Street, Toronto, ON M5G 1L7 Canada

**Keywords:** Metabolic syndrome, Preclinical research

## Abstract

**Background/objectives:**

Low-grade chronic inflammation in visceral adipose tissue and the intestines are important drivers of obesity associated insulin resistance. Bioactive compounds derived from plants are an important source of potential novel therapies for the treatment of chronic diseases. In search for new immune based treatments of obesity associated insulin resistance, we screened for tissue relevant anti-inflammatory properties in 20 plant-based extracts.

**Methods:**

We screened 20 plant-based extracts to assess for preferential production of IL-10 compared to TNFα, specifically targetting metabolic tissues, including the visceral adipose tissue. We assessed the therapeutic potential of the strongest anti-inflammatory compound, indigo, in the C57BL/6J diet-induced obesity mouse model with supplementation for up to 16 weeks by measuring changes in body weight, glucose and insulin tolerance, and gut barrier function. We also utilized flow cytometry, quantitative PCR, enzyme-linked immunosorbent assay (ELISA), and histology to measure changes to immune cells populations and cytokine profiles in the intestine, visceral adipose tissue (VAT), and liver. 16SrRNA sequencing was performed to examine gut microbial differences induced by indigo supplementation.

**Results:**

We identifed indigo, an aryl hydrocarbon receptor (AhR) ligand agonist, as a potent inducer of IL-10 and IL-22, which protects against high-fat diet (HFD)-induced insulin resistance and fatty liver disease in the diet-induced obesity model. Therapeutic actions were mechanistically linked to decreased inflammatory immune cell tone in the intestine, VAT and liver. Specifically, indigo increased Lactobacillus bacteria and elicited IL-22 production in the gut, which improved intestinal barrier permeability and reduced endotoxemia. These changes were associated with increased IL-10 production by immune cells residing in liver and VAT.

**Conclusions:**

Indigo is a naturally occurring AhR ligand with anti-inflammatory properties that effectively protects against HFD-induced glucose dysregulation. Compounds derived from indigo or those with similar properties could represent novel therapies for diseases associated with obesity-related metabolic tissue inflammation.

## Introduction

Obesity is a major risk factor for type 2 diabetes and is associated with low-grade chronic inflammation [1]. The precursor to type 2 diabetes, known as insulin resistance (IR), can develop through multiple pathways, but the role of the immune system inside metabolic tissues and the intestine is an important contributor to this disease [2–6]. High-fat diet (HFD) changes the composition of the gut microbiota, leading to dysbiosis, which contributes to inflammatory immune changes in the bowel [7]. Leaked intestinal luminal components, such as lipopolysaccharides (LPS), reach the visceral adipose tissue (VAT) and other metabolic tissues including the liver, and promote local inflammation and insulin insensitivity [8–10]. During obesity, the expansion of VAT leads to adipocyte death resulting in the formation of immune cell crown-like structures (CLS) composed of accumulating immune cells [11, 12]. Pro-inflammatory cytokine secretion, including interferon (IFN)γ from CD8+ T cells and CD4+ Th1 T cells, and TNFα, interleukin (IL)-6 and IL-1β from recruited inflammatory (M1-like) polarized macrophages, can directly inhibit the insulin signaling pathway in tissues [11, 13, 14]. Regulatory T cells (Tregs) and resident anti-inflammatory (M2-like) polarized macrophages that produce factors such as IL-10 help to maintain insulin sensitivity but are reduced within the VAT during obesity [15, 16].

The gut is home to the largest component of the body’s immune system known as the gut-associated lymphoid tissue. During homeostatic conditions, immune cells of the gut function to maintain the integrity of the intestinal barrier, promote tolerance to food antigens and commensal bacteria, and protect against invasive pathogens. Tregs and the production of IL-10 promotes mucin production by Goblet cells, and tolerance to the gut flora and dietary proteins [17, 18]. In addition, RORγt+ innate lymphoid cells (ILCs) and IL-22 producing CD4+ T cells can produce IL-22 to support the barrier function of intestinal epithelial cells (IECs) [19, 20]. One such mechanism is through the production of antimicrobial peptides (AMPs) by Paneth cells that regulate the gut microbiota and intestinal barrier integrity [21, 22].

In contrast, during obesity, hyperglycemia and pro-inflammatory mediators can disrupt intestinal barrier function, increase intestinal permeability, and lead to altered gut immunity with low levels of chronic inflammatory change [3, 23]. This compromised intestinal barrier results in bacterial product leakage and metabolic tissue inflammation, including increases in TNFα and reductions in IL-10 in VAT [2, 24]. In the gut, diet-induced obesity (DIO) in mice and humans leads to a Th1 response with increased IFNγ production coupled with a reduction in Th17 cells [4–6]. Furthermore, decreased intestinal ILC3s and IL-22-producing CD4+ T cells also occur in HFD-fed mice which may result in less IL-22 production, worsening intestinal barrier function [3, 25]. Administration of exogenous IL-22 in HFD-fed mice improves the gut barrier, reduces LPS leakage and alleviates IR [25, 26]. Overall, IL-22 production is critical for intestinal homeostasis and may be an important therapeutic target for obesity and IR.

The aryl hydrocarbon receptor (AhR) is a transcription factor first identified for mediating the toxicity of environmental pollutants. Since then, AhR has also been discovered to be important for multiple physiological mechanisms, especially in the regulation of intestinal homeostasis. In metabolic syndrome, the production of AhR agonists by the gut microbiota is reduced and restoring AhR signaling induces beneficial metabolic effects [4, 27]. AhR-dependent production of IL-22 and AhR itself can enhance IEC proliferation and production of AMPs [20]. AhR can directly regulate IL-22 gene expression and cytokine production within immune cells or modulate ILC3 and IL-22 producing CD4+ T cell development [22]. AhR agonists have also been shown to directly induce IL-10 responses in myeloid cells to counter autoimmune disorders such as lupus [28]. Thus, new therapeutics that target AhR may improve HFD-induced intestinal barrier dysfunction, downstream inflammatory changes and overall obesity-related IR.

To look for novel natural therapeutics for IR, we screened 20 plant-derived compounds or crude extracts, which were previously reported to induce anti-inflammatory, immune-modulatory or thermogenesis effects, and assessed their influence on IL-10 and TNFα in VAT stromal vascular cell (SVC) extracts of HFD-fed mice. The output of our screening identified indigo (C_16_H_10_N_2_O_2_), the active ingredient of *Indigo Naturalis*, to markedly increase IL-10 and IL-22 production by immune cells in VAT and the small intestine. Indigo is known to be a potent AhR ligand composed of an indole ring structure similar to other AhR ligands, including tryptophan metabolites and another natural compound, indirubin [29]. *Indigo Naturalis* has been used to treat chronic diseases such as psoriasis and ulcerative colitis, but its effect on obesity and IR is unknown [30, 31]. Due to its effects in modulating intestinal immune and barrier function and other chronic inflammatory diseases, we hypothesize that indigo might have beneficial effects in obesity-related IR. Here, we show that indigo improves gut barrier function, VAT inflammation, and IR through the activation of the AhR, associated with IL-22 production in the small intestine and IL-10 responses in metabolic tissues. Targeting the AhR may represent an important class of potential therapeutics for obesity-related metabolic complications such as IR.

## Materials and methods

### Plant-derived compounds

Indigo, Aliso B acetate, gentiopicroside, peimine, perillaldehyde, wedelolactone, bergapten, picroside I, berberine, cinnamaldehyde, ephedrine, p-synephrine, 6-shogao, and 6-gingerol were purchased from Sigma-Aldrich. Phellodendrine was purchased from Abcam. Baicalein, baicalin, wogonin, wogonoside, and *Ephedra sinica* were kindly provided by Y.L. Leu (Chang Gung University, Taiwan). *Scutellaria baicalensis* (precursor of Baicalein, baicalin, wogonin, wogonoside) and *Ephedra sinica* were supplied and authenticated by Department of Pharmacy Services, Chang Gung Memorial Hospital at Taoyuan, Taiwan. See Supplementary Information for more details on compound preparation.

### Mice and diet

We purchased C57BL/6J and B6.129S7-*Rag1*^*tm1Mom*^/J mice from Jackson Laboratory. In our mouse model of DIO, wild-type (WT), age-matched, male mice were randomly assigned to receive either HFD (Research Diets, 60% kcal fat) or HFD supplemented with indigo (HFD-Indigo) by Research Diets Inc. at a dose of 300 mg/kg/day based on average food intake per day starting at 6 weeks of age for up to 16 weeks of diet. Similarly, indigo was also mixed into normal chow diet (NCD-Indigo) by Envigo Teklad diets at a dose of 300 mg/kg/day. Mice were bred in a pathogen-free, temperature-controlled environment on a 12 h light and dark cycle. All animal studies were performed under the approval of Animal User Protocols by the Animal Care Committee at the University Health Network. See Supplementary Information for full details on study design.

### Metabolic cage studies

We placed mice in automated metabolic cages (Oxymax Systems, Columbus Instruments) for 48 h with airflow held constant at 0.5 L/min and monitored for food and water intake [32]. See Supplementary Information.

### Body temperature

Rectal temperature of mice was measured at 14 weeks of HFD at 10:00 AM.

### Metabolic studies

All mice were weighed every 2 weeks. After 10 weeks of diet, fasting blood glucose and insulin levels were measured (Crystal Chem Inc. ELISA). For glucose tolerance test (GTT), fasted (16 h) mice received 1.5 g/kg glucose i.p. injection. For insulin tolerance test studies, mice received 0.75 U/kg of human regular insulin (Eli Lilly).

### Histology

We fixed VAT and liver from mice for 48 h in 10% buffered formalin before processing and H&E staining. See Supplementary Information.

### Tissue immune cell isolation

Immune cells were isolated from VAT, small intestine and spleen as previously described [33, 34]. Cytokine measurements (IL-10, TNFα, and IL-22) in the supernatants of immune cells or small intestinal tissue explants of HFD mice were assessed by ELISA. See Supplementary Information.

### In vitro IL-22-producing CD4+ T-cells differentiation

Mesenteric lymph nodes from 14 weeks HFD-fed mice were isolated and naïve CD4+ T cells were purified. After culture under a Th17/Th22 differentiation protocol (BioLegend) with indigo or DMSO for 4 days, supernatants were collected to assess IL-22 levels. See Supplementary Information.

### Flow cytometry

Cells were stained with commercial fluorophore-conjugated primary antibodies listed in the Supplementary Methods using recommended dilutions from the supplier (BioLegend). See Supplementary Information.

### RNA isolation and quantitative real time-PCR

Primer sets are listed in Supplemental Table 1. Changes in gene expression were calculated by the ∆∆CT method using the equation 2^−△△CT^. Expression of the housekeeping gene, *Actb*, did not change between groups and results are shown as fold changes relative to the control group. See Supplementary Information.

### Triglyceride assays

Serum and liver triglycerides were measured by using the Triglyceride Quantification Colorimetric kit, following manufacturer’s instructions (Sigma).

### Liver function tests

Serum alanine transaminase (ALT) was measured by Bio-Rad Liquid Assayed Multiqual (The Centre for Phenogenomics—Mount Sinai Hospital).

### Small intestine tissue explant culture

Small intestines were cut into 1 cm segments [35] and cultured in RPMI 1640 containing 10% FBS, indigo (50 μM) dissolved in dimethyl sulfoxide (DMSO), FICZ (300 nM, AhR agonist used as a positive control; Sigma), or DMSO control (at 0.05% final concentration), and/or IL-1β 40 ng/mL stimulation in 24-well flat-bottom plates. Tissues were cultured at 37 °C in a humidified atmosphere of 5% CO_2_ and 95% air. FICZ is a known potent and high affinity endogenous AhR ligand formed from its precursor indole-3-acetaldehyde via oxidation of tryptophan by intracellular oxidants and enzymatic deamination of tryptamine [36].

### Gut permeability assays

Gut permeability was measured by concentrations of orally-gavaged macromolecule 4-kDa fluorescein isothiocyanate-dextran (FD4) in plasma 4 h post-gavage. See Supplementary Information.

### Anti-LPS IgG antibody measurements

We measured mouse serum anti-LPS IgG antibody levels with a commercially available kit (Chondrex).

### Statistical analysis

Statistical significance between two means, unless stated otherwise, was assessed with an unpaired, two-sided *t* test. Dataset normality was confirmed by Shapiro–Wilk normality test. For statistical analysis of gut microbial sequencing, see Supplementary Information. In figure legends, where specified, the number of biological replicates or experiments is listed as the *n* value, followed by the number of pooled mouse samples. All data represent mean ± SEM. Statistical significance was set at *p* < 0.05. Statistical significance is denoted by ^*^p < 0.05, ^**^p < 0.01, ^***^p < 0.001.

## Results

### Chemical screen of plant derivatives identifies Indigo as a strong inducer of IL-10 and IL-22 production in HFD-induced immune cells

**Fig. 1:**
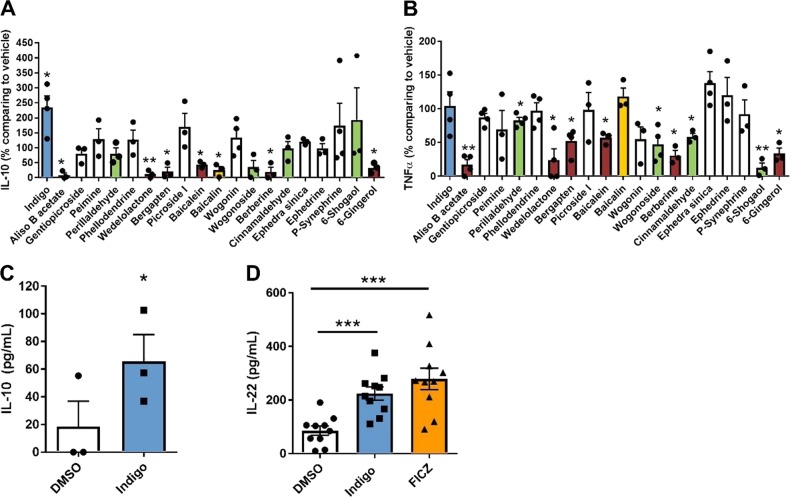
Chemical screen of plant derivatives identifies indigo as an inducer of anti-inflammatory cytokines, including IL-10 and IL-22, in HFD-induced immune cells and small bowel tissue explants. **a**, **b** Cytokine production, either IL-10 or TNF-α, in the VAT SVC of HFD mice treated with drug candidates or their vehicle (DMSO, EtOH, or water) for 3 days (IL-10: *n* = 4 experiments in indigo, wogonin, p-synephrine; *n* = 3 in other drugs pooled from 15 mice per experiment. TNF-α: *n* = 4 experiments in indigo, Aliso B acetate, gentiopicroside, perillaldehyde, phellodendrine, wedelolactone, bergapten, wogonoside, *Ephedra sinica*; *n* = 3 in other drugs pooled from 15 mice per experiment). Drug concentration: ephedrine is 10 μM; Indigo, gentiopicroside, peimine, perillaldehyde, phellodentrine, bergapten, picroside I, berberine and cinnamaldehyde 50 μM; Aliso B acetate, wedelolactone, baicalein, baicalin, wogonin, wogonoside, p-synephrine, 6-shogaol and 6-gingerol are 250 μM; *Ephedra sinica* is 1 μg/mL. Different bar colors represent indigo (blue), drugs decreasing IL-10 and TNFα (red), drugs decreasing IL-10 only (yellow), and drugs decreasing TNFα only (green). **c** IL-10 of cultured intestinal lamina propria immune cells supernatant after 72 h treated in vitro with 50 μM indigo and 0.05% DMSO under lipopolysaccharide (LPS) stimulation (*n* = 3/group, paired *t* test). **d** IL-22 in small bowel tissue explant culture supernatant of HFD-fed mice treated in vitro with 0.05% DMSO, 50 μM Indigo, 300 nM FICZ for 24 h (*n* = 10/group). Data in bar graphs represent mean ± SEM. *p < 0.05, **p < 0.01, ***p < 0.001

We developed an immunological screening assay to assess the effects of multiple plant-derived compounds on VAT inflammation, a critical site of immunological influence on whole-body IR. We utilized 20 plant-derived compounds, previously known to have immune modulating properties, in HFD-induced VAT SVC. The output of the screening consisted of cytokine readouts known to modulate IR, including TNFα and IL-10. The 20 plant-derived or crude extracts in this screen consisting of indigo, aliso B acetate, gentiopicroside, peimine, perillaldehyde, phellodendrine, wedelolactone, bergapten, picroside I, baicalein, baicalin, wogonin, wogonoside, berberine, cinnamaldehyde, *Ephedra sinica*, ephedrine, p-synephrine, 6-shogaol, and 6-gingerol were tested in three different doses according to available literature and compared to the vehicle in the production of these cytokines by VAT SVC of mice fed 14 weeks of HFD (Figs. S1 and S2). We sought to identify target candidates that could either (1) increase IL-10 without increasing TNFα or (2) decrease TNFα without decreasing IL-10. The result of our screen excluded six plant derivatives as target candidates as they decreased TNFα levels and IL-10 simultaneously, including aliso B acetate, wedelolactone, bergapten, baicalein, berberine, and 6-gingerol (Fig. 1a, b). Interestingly, indigo was the only compound which markedly increased IL-10 in VAT SVC, without altering levels of TNFα (Fig. 1a). Four plant derivatives, perillaldehyde, wogonoside, cinnamaldehyde, and 6-shogaol reduced levels of TNFα without decreasing IL-10, marking these compounds as potential immunomodulatory therapeutics for metabolic disease (Fig. 1b). The roles of wogonoside, cinnamaldehyde, and 6-shogaol or their related compounds have been previously studied in the context of metabolic disease, however, their immunomodulatory properties could also be of interest for further study [37–39]. Since indigo was observed to be a strong inducer of IL-10 and its documented effect as a potent AhR ligand [29] with the potential to impact metabolic disease, we identified indigo as our target of interest for this study. Since AhR activation is important in the induction of another IL-10 family cytokine, IL-22, in the small intestine [22], we next assessed the effect of indigo on IL-10 and IL-22 levels in the intestine of HFD-fed mice. Addition of indigo to cultured small intestinal immune cells increased IL-10 production (Fig. 1c). Furthermore, levels of IL-22 from small bowel tissue explants were also increased with indigo treatment in vitro (Fig. 1d). Similar effects were seen upon addition of a potent AhR agonist, FICZ, raising the possibility that indigo may also exert its protective effects through an AhR pathway (Fig. 1d). Thus, due to its strong anti-inflammatory effects in the VAT and small intestine, indigo was identified to be an ideal plant derivative candidate to study in the context of immune modulation in obesity-related IR.

### Indigo supplementation alleviates diet-induced metabolic disease in HFD-fed mice

**Fig. 2:**
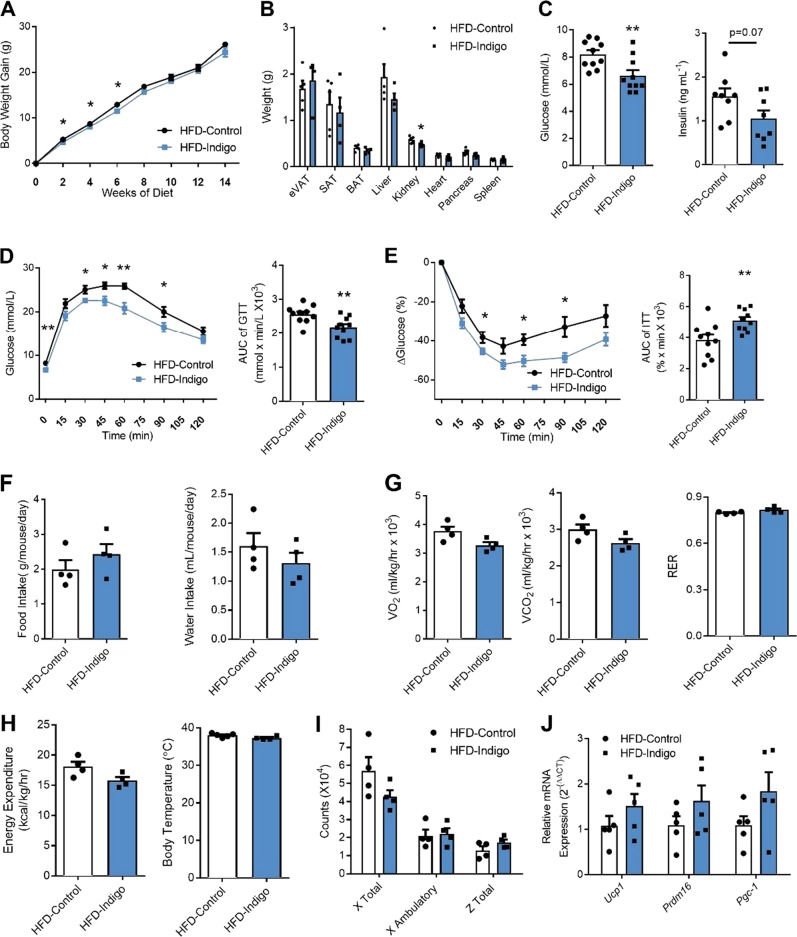
Indigo improves metabolic parameters associated with diet-induced obesity. **a** Body weight gain of HFD and HFD-Indigo (300 mg/kg/day)-fed C57BL/6J mice over time, starting at 6 weeks of age (*n* = 10/group). **b** Organ weights of HFD-Control and HFD-Indigo mice (*n* = 5 in HFD-Control, *n* = 4 in HFD-Indigo, *p* = 0.18 for liver). **c** Fasting glucose (left), fasting insulin (right) (*n* = 10 mice for glucose, *p* = 0.07 for insulin, *n* = 8 mice). **d**, **e** Glucose tolerance test (GTT, **d**, left), AUC of GTT (**d**, right), insulin tolerance test (ITT, **e**, left) and AUC of ITT (**e**, right) of mice after 10 weeks of HFD or HFD-Indigo (300 mg/kg/day) (*n* = 10 mice for GTT, *n* = 10 mice for ITT). **f** Food intake (left) and water intake (right) (*n* = 4 mice/group). **g**–**i** Metabolic cage analysis including oxygen consumption (**g**, left), carbon dioxide production (**g**, middle), respiratory exchange ratio (RER) (**g**, right), energy expenditure (**h**, left), body temperature (**h**, right) and activity (**i**) of HFD-Control and HFD-Indigo mice (*n* = 4 mice/group for metabolic cage analysis, *n* = 5 HFD-Control and *n* = 4 HFD-Indigo mice for body temperature). (**j**) mRNA expression of thermogenesis related genes (*Ucp1*, *Prdm16*, *Pgc-1*) in brown adipose tissue (*n* = 5/group). Data in bar graphs represent mean ± SEM. ^*^p < 0.05, ^**^p < 0.01, ^***^p < 0.001

To determine the effects of indigo in obesity-related IR  in vivo, we fed mice at 6 weeks of age with either, NCD, NCD-Indigo, HFD, or HFD-Indigo. Minimal differences were seen in body weight gain between 2 and 6 weeks of HFD-Indigo feeding compared to HFD, but this effect was lost after 8 weeks of diet (Fig. 2a). Organ weights showed a slight reduction in kidney weights in HFD-Indigo fed mice after 14 weeks of diet (Fig. 2b). Fasting glucose (Fig. 2c, left) improved in HFD-Indigo fed mice, though fasting insulin only showed a trending decrease (Fig. 2c, right). HFD-Indigo mice also displayed ameliorated glucose tolerance (Fig. 2d), and insulin tolerance (Fig. 2e) compared to controls after 10-weeks HFD. Indigo supplementation in NCD fed mice minimally improved glucose tolerance, but did not affect body weight or insulin tolerance (Fig. S3). To measure changes to energy parameters, we placed these mice in metabolic cages to measure changes to energy activity. We observed no differences in food intake (Fig. 2f, left), water intake (Fig. 2f, right), oxygen consumption (VO_2_) (Fig. 2g, left), carbon dioxide production (VCO_2_) (Fig. 2g, middle), respiratory exchange ratio (RER) (Fig. 2g, right), energy expenditure (Fig. 2h, left), body temperature (Fig. 2h, right), or activity (Fig. 2i) between mice given HFD-Indigo or HFD alone. Consistent with the lack of increased energy expenditure, we did not detect significant changes in the expression of genes linked to adipose tissue thermogenesis (*Ucp1*, *Prdm16*, *PGC-1α*) in brown adipose tissue (Fig. 2j) [40]. Overall, our results indicate that indigo supplementation has therapeutic effects on glucose homeostasis in the setting of DIO.

### Amelioration of immune-mediated inflammatory changes in the intestine, VAT, and liver in HFD-Indigo fed mice

**Fig. 3:**
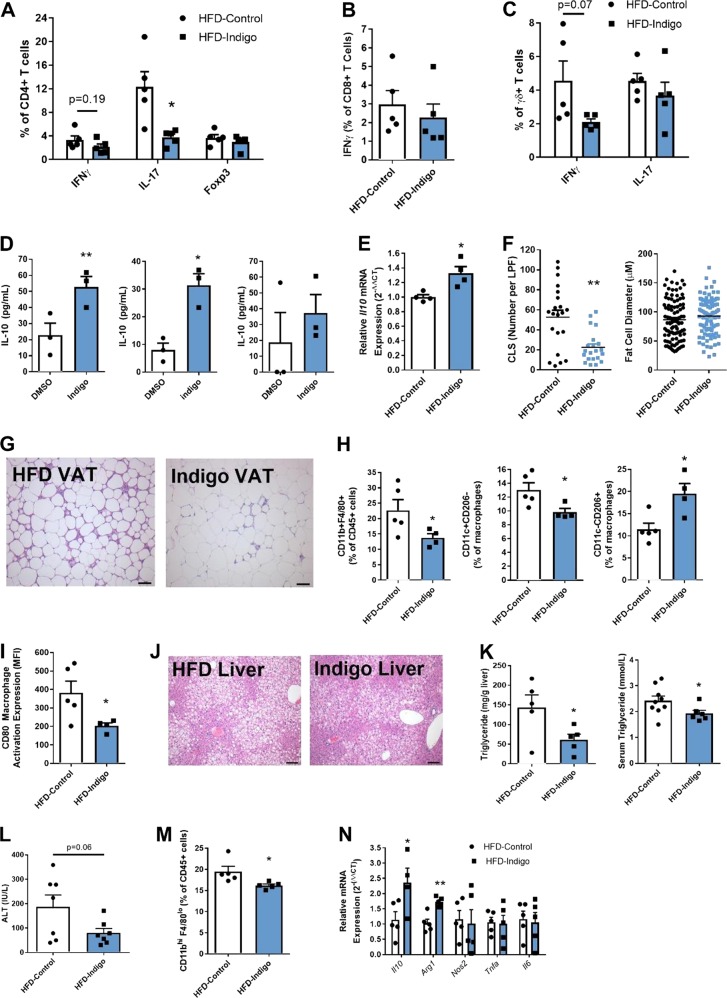
Amelioration of immune-mediated inflammatory changes in the intestine, VAT, and liver in HFD-Indigo fed mice. **a**–**c** Intracellular staining of cytokines and Foxp3 transcription factor in the small intestine lamina propria (LP) CD4+ (including Foxp3+ regulatory T cells), CD8+, and γδ+ T cell populations of mice after 14 weeks of HFD or HFD-Indigo (*n* = 5 mice/group). **d** In vitro IL-10 level in VAT SVC total immune cells (left), CD11b positively selected immune cells (middle), and CD11b negatively selected immune cells (right) treated with DMSO and 50 μM indigo for 3 days (*n* = 3/group with 2 pooled mice). **e** *Il10* mRNA expression of VAT whole tissue from HFD-Control and HFD-Indigo mice (*n* = 4/group). **f** Number of VAT “crown-like structures (CLS)” per 100× low-power field (left), and relative fat cell diameter (right) of mice after 14 weeks of HFD (CLS fields counted from *n* = 3 mice; relative fat cell diameter counted from one representative mouse, ^**^p = 0.0028 Mann–Whitney test). **g** Representative histology of VAT after 16 weeks of HFD or HFD-Indigo mice (H&E stain, scale bar 100 µm). **h**, **i** Flow cytometry analysis of the percentage of CD11b+ F4/80+ macrophages (**h**, left), percentage of CD11c+ CD206− M1-like macrophages subset (**h**, middle), percentage of CD11c- CD206+ M2-like macrophages subset (**h**, right), and CD80 macrophage activation expression (**i**), in VAT of mice after 14 weeks of HFD or HFD-Indigo (*n* = 5 in HFD-Control, *n* = 4 in HFD-Indigo mice, ^*^p = 0.0317 Mann–Whitney test for M1-like macrophages). **j** Representative histology of livers after 16 weeks of HFD or HFD-Indigo mice (H&E stain, scale bar 100 µm). (**k**, left) Liver triglyceride content after 16 weeks of HFD or HFD-Indigo mice (*n* = 5/group). (**k**, right) Serum triglyceride after 14 weeks of HFD or HFD-Indigo mice (*n* = 9 in HFD-Control, *n* = 6 in HFD-Indigo mice). **l** Serum levels of alanine transaminase (ALT) after 16 weeks of HFD or HFD-Indigo mice (*n* = 7/group). **m** Flow cytometry analysis of percentage of CD11b^hi^, F4/80^lo^ recruited hepatic macrophages in the liver of mice after 16 weeks of HFD or HFD-Indigo (*n* = 5/group). **n** mRNA expression of *Il10*, M2 marker (*Arg1*) and M1 markers (*Nos2*, *Tnfa*, *Il6*) in the liver (*Il10*: *n* = 4 in HFD-Indigo mice, others *n* = 5/group). Data in bar graphs represent mean ± SEM. ^*^p < 0.05, ^**^p < 0.01

We next sought to determine the mechanisms behind the amelioration of IR by indigo. Given that low-grade chronic inflammation of the intestine and metabolic tissues is central to the pathogenesis of obesity-related IR, we first assessed inflammatory changes in such tissues in indigo fed mice. HFD-Indigo fed mice exhibited a reversal of the local pro-inflammatory immune shift induced by HFD feeding in the small bowel that has been previously reported [3]. Within the small intestine, HFD-Indigo mice displayed a decrease in both the absolute number and percentage of IL-17 producing CD4+ T cells, a decrease in absolute number of IFNγ producing CD4+ T cells, and no alterations in the Foxp3+ regulatory T cells (Figs. 3a, S4). In addition, the numbers but not percentage of IFNγ and IL-17 secreting γδ+ T cells were also decreased by indigo feeding (Figs. 3c, S4). Both the percentage and amount of IFNγ-secreting CD8+ T cells were unchanged (Figs. 3b,  S4). Splenic T-cell subsets were also not altered by indigo feeding (Fig. S5). Indigo treatment in HFD-fed mice also showed increasing mRNA expression of M2-like macrophage marker *Arg1* in the small intestine (Fig. S6).

As the adipose tissue and liver are key metabolic tissues impacted by obesity-related IR, we next determined whether indigo had immune-modulatory functions on cells or tissue from these sites. In vitro treatment of total and CD11b+ purified VAT SVC immune cells from HFD-fed mice with 50 μM indigo significantly increased IL-10 secretion (Fig. 3d left and middle), though increases in CD11b− cells did not reach significance (Fig. 3d right), suggesting that indigo influences IL-10 production by VAT myeloid cells. In vivo, HFD-Indigo fed mice increased VAT *Il10* mRNA expression compared to HFD-fed controls (Fig. 3e) and had an induction in the expression of the AhR target gene, Cytochrome P450 1A1 (*Cyp1a1*) (Fig. S7), suggesting that indigo may induce VAT IL-10 production via AhR activation. Furthermore, HFD-Indigo mice displayed a reduction in the number of CLS in the VAT (Fig. 3f left and G), though overall adipocyte size was not significantly altered (Fig. 3f, right). Using flow cytometry, we identified that HFD-Indigo mice had decreased percentage of total immune cells as CD11b+ F4/80+ macrophages in VAT without alterations to macrophages in the spleen (Fig. 3h left and S8). The balance of macrophage subsets within the VAT was shifted towards reduced classical M1-like macrophages (CD11c+ CD206−) and increased alternative anti-inflammatory M2-like macrophages (CD11c− CD206+) (Fig. 3h middle and right). Moreover, macrophage expression of the activation marker, CD80, was decreased in HFD-Indigo fed mice (Fig. 3i). In the liver, HFD-Indigo mice had reduced steatosis as shown by histology and decreased triglyceride content compared to HFD-control mice (Fig. 3j, k, left). Serum triglyceride levels were also reduced in HFD-Indigo fed mice compared to HFD controls (Fig. 3k, right). To further assess liver function, we measured serum levels of alanine aminotransferases (ALT), which displayed a trending reduction in HFD-Indigo mice suggesting that indigo supplementation may indirectly improve liver function (Fig. 3l). Flow cytometry analysis revealed decreased percentage and number of CD11b^hi^, F4/80^lo^ recruited hepatic macrophages in the liver of HFD-Indigo mice (Fig. 3m, S9). In addition, similar to adipose tissue, HFD-Indigo mice showed increased liver mRNA expression of *Il10* and M2 marker (*Arg1*) but no difference in M1 markers (*Nos2, Tnfa, Il6*) (Fig. 3n). Together, indigo supplementation has beneficial immune-modulatory effects in the intestine, VAT, and liver contributing to the overall improvement in whole-body glucose homeostasis.

### Indigo increases intestinal IL-22 production and improves intestinal barrier function during HFD

**Fig. 4:**
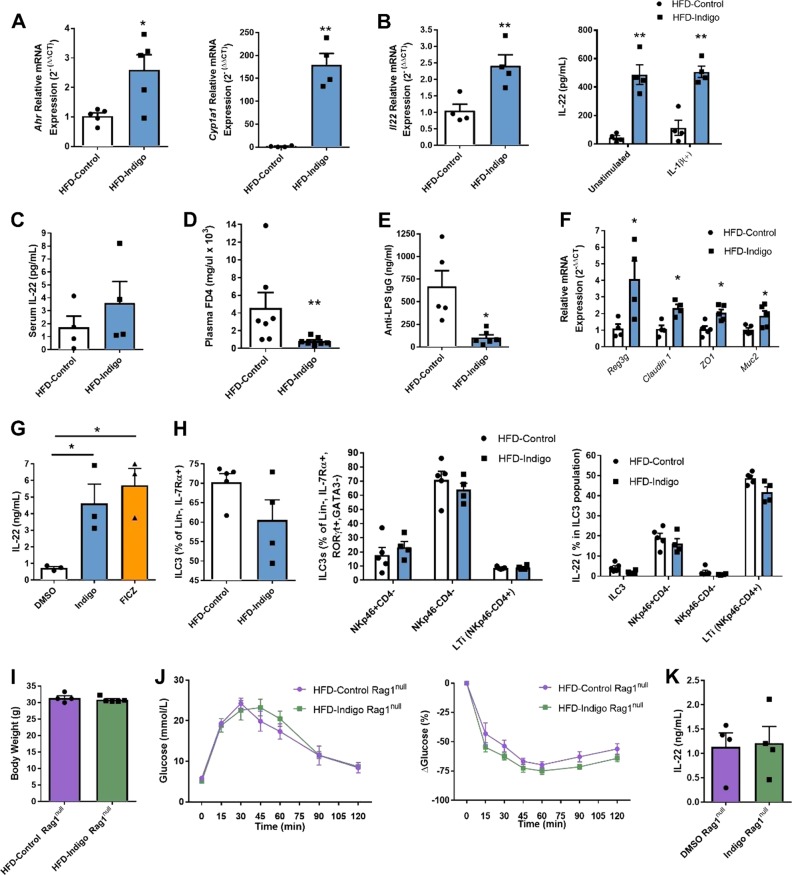
Indigo increases intestinal IL-22 and improves intestinal barrier function during HFD. **a** AhR pathway genes, *Ahr* and *Cyp1a1*, mRNA expression in small intestine (*n* = 5/group). (**b**, left) *Il22* mRNA expression of small bowel whole tissue from HFD-Control and HFD-Indigo mice (*n* = 4/group). (**b**, right) IL-22 in small bowel tissue explant with or without IL-1β 40 ng/mL stimulation in HFD-Control and HFD-Indigo wild-type mice (*n* = 5/group). **c** Serum IL-22 in HFD-Control and HFD-Indigo mice (*n* = 4/group). **d** Plasma FD4 concentration after 4 h following oral gavage in HFD-Control, and HFD-Indigo after 13 weeks of diet (*n* = 7/group, ^**^p = 0.007 Mann–Whitney test). **e** Serum anti-LPS IgG levels of age-matched HFD-Control and HFD-Indigo mice after 14 weeks HFD (*n* = 5 in HFD-Control, *n* = 6 in HFD-Indigo mice). **f** *Reg3g*, *claudin1*, *ZO1*, and *Muc2* mRNA expression of small bowel whole tissue from HFD-Control and HFD-Indigo mice (*Reg3g*, *claudin1*: *n* = 4/group; *ZO1*, *Muc2*: *n* = 5/group). **g** IL-22 in differentiated IL-22 producing CD4+ T cells from mesenteric lymph nodes of WT HFD mice in vitro treated with DMSO, Indigo 50 μM, and 300 nM FICZ for 4 days (*n* = 3/group). **h** Flow cytometry analysis of total ILC3s, ILC3 subsets and their IL-22 secretion in LP of mice after 15 weeks of HFD or HFD-Indigo (300 mg/kg/day) (*n* = 5 in HFD-Control, *n* = 4 in HFD-Indigo mice). **i**, **j** Body weights (**i**), GTT (**j**, left), and ITT (**j**, right) of Rag1^null^ mice after 10 weeks of HFD or HFD-Indigo (300 mg/kg/day) (*n* = 4 in HFD-Control, *n* = 5 in HFD-Indigo Rag1^null^ mice). **k** IL-22 in the small bowel tissue explant culture supernatant of Rag1^null^ mice fed with HFD for 14 weeks ex vivo stimulated with IL-1β 40 ng/mL and treated with 0.05% DMSO and 50 μM indigo for 24 h (*n* = 4/group). Data in bar graphs represent mean ± SEM. ^*^p < 0.05, ^**^p < 0.01

Since indigo is thought to be an AhR ligand, it is possible that the beneficial effects of indigo seen in metabolic and intestinal tissue inflammation are associated with AhR-dependent modulation of the intestinal barrier via IL-22 [20, 41]. To first examine the effects of indigo as an AhR ligand and its downstream effects of AhR activation, we assessed mRNA expression of *Ahr* and its classical target gene *Cyp1a1* in the small intestine of HFD-Indigo mice compared to mice fed HFD alone. Indeed, HFD-Indigo fed mice displayed increased *Ahr* and *Cyp1a1* gene expression in whole small bowel tissue signifying augmented intestinal AhR activity in these mice (Fig. 4a). We next investigated whether HFD-Indigo could alter IL-22 production in the small bowel in vivo. In HFD-Indigo fed mice, the upregulation of small intestinal *Il22* gene expression in whole tissue homogenates concurred with increased IL-22 levels in tissue explants cultured ex vivo with or without IL-1β stimulation (Fig. 4b). Indigo supplementation, however, did not significantly affect serum IL-22 levels (Fig. 4c).

Due to the critical role for local IL-22 in promoting gut barrier integrity, we hypothesized that key mediators of intestinal barrier function including AMPs, tight junction proteins and mucous production, may be altered with indigo supplementation in obese mice [26]. Indigo treatment improved intestinal paracellular permeability in vivo, as measured by fluorescence FD4 assay (Fig. 4d). Since impaired barrier function can increase systemic gut-derived microbial products, such as LPS, also known as metabolic endotoxemia [9], we measured serum anti-LPS IgG levels. Circulating IgG responses to LPS were markedly diminished in indigo-treated mice (Fig. 4e). In addition, gene expression of the antimicrobial peptide (regenerating islet-derived 3gamma, *Reg3*g), tight junction proteins (*claudin 1* and *ZO1)*, and goblet cell marker (*Muc2)* were increased in the small intestine of HFD-Indigo fed mice (Fig. 4f), suggesting a possible mechanism for indigo to influence gut permeability. Overall, these results demonstrate effects of indigo supplementation to increase IL-22 production and ameliorate HFD-induced gut barrier dysfunction.

We next examined the effects of indigo on IL-22 producing immune cell types in small bowel during HFD feeding. Since the major sources of AhR-dependent IL-22 in the small intestine are ILC3s and Th17/Th22 cells, we further investigated how indigo supplementation influenced these immune cell populations. To test if the increase in IL-22 secretion was attributed to T cells, we differentiated Th17/Th22 cells from mesenteric lymph nodes of HFD mice in vitro and added indigo and FICZ. Levels of IL-22 in Th17/Th22 cells were markedly increased by the treatment of indigo similar to FICZ treatment compared to DMSO alone (Fig. 4g). Interestingly, indigo treatment showed no effects on the amount of total ILC3, ILC3 subsets, and their secretion of IL-22 in the small intestine (Fig. 4h). To evaluate if the effect of indigo on IL-22 production was mainly from adaptive immune cells, we fed HFD-Indigo to Rag1^null^ mice which are deficient in adaptive T and B cells but retain ILCs. We treated 6-week-old Rag1^null^ mice with HFD-Indigo compared with HFD alone for 10 weeks. The protective effects previously detected in indigo supplementation in wild-type mice were absent or reduced in Rag1^null^ mice as we observed no significant differences in body weight, glucose tolerance, or IR (Fig. 4i, j), suggesting that the beneficial effects of indigo utilize components of the adaptive immune system. Changes to the levels of IL-22 were also not seen in small intestine tissue explants of HFD Rag1^null^ mice treated with indigo compared to DMSO controls ex vivo (Fig. 4k) or in small bowel explants from HFD-Indigo Rag1^null^ mice (Fig. S10). Thus, based on the model systems used, the beneficial effects of indigo in glucose homeostasis and gut immune and barrier function likely preferentially target IL-22 production in intestinal and gut lymphoid tissue related T cells.

Due to the known role for IL-22 in shaping the gut microbiota [35, 42], we investigated if Indigo supplementation could alter the composition of the gut microbiota. We performed 16S rRNA sequencing of the small intestinal luminal contents in HFD-Indigo and HFD mice (Fig. S11). HFD-Indigo mice did not display differences in microbial richness or evenness as shown by the Shannon Diversity Index and observed genera analysis. Overall, indigo supplementation increased proportions of Lactobacillus, a bacterial genus that is essential to tryptophan catabolism, while also decreasing Akkermansia genera in HFD-fed mice which supports the observations made by other groups in IL-22 deficient mice and AhR−/− mice, respectively [42–44].

## Discussion

Here, we have shown that an AhR ligand, indigo, has therapeutic potential to modulate inflammatory tone in obesity-related IR. The role of the AhR is multifaceted and varies largely with ligand type, tissue specificity, and disease models. For instance, in experimental autoimmune encephalomyelitis, two potent AhR agonists, TCCD and FICZ, have opposing effects in which the former decreases Th17 differentiation while the latter increases Th17 cells [45]. Within the intestine, AhR is necessary for normal Th17 development [46], however, in colitis, TCCD may decrease Th17 differentiation [47]. Thus, specific AhR ligands and their specific role in various tissues and disease models must be examined. In obesity, studies have demonstrated that AhR mediates DIO and its whole-body deletion in mice protects from diet-induced adiposity and metabolic disorders [48, 49], while AhR has also been reported to have a protective role in HFD-induced hepatic steatosis [50]. In a recent study, metabolic syndrome was associated with reduced capacity of gut bacteria to metabolize tryptophan into AhR ligands in mice and humans [27]. Thus, the identification of new or repurposed AhR ligands with the capacity to alter obesity-related metabolic disease represents an important avenue of new therapy development. Following a chemical screen of 20 plant-derived candidates with immune modulating properties, we identified indigo, a specific AhR agonist, as an effective anti-inflammatory therapy for DIO related IR in mice in vitro and in vivo.

Indigo supplementation at a dose of 300 mg/kg/day was able to alter systemic glucose metabolism during HFD feeding, without altering energy expenditure. We have previously shown that HFD enhances a pro-inflammatory shift in gut immune cell populations, characterized by increased IFNγ-producing Th1 cells and CD8+ T cells [3]. In this study, we observed beneficial effects of indigo on gut immune function during HFD feeding, associated with reduced amounts of IFNγ-releasing immune cells. Cytokines such as IFNγ can compromise gut barrier integrity, allowing for gut microbial product leakage and worsening of inflammation in the intestine [51]. This finding is reinforced by previous reports that AhR plays an important role in the immune system by downregulating IFNγ and IFNγ+ CD4+ T cells are substantially increased in AhR−/− mice [52]. Despite the potential protective role of IL-17 in maintaining intestinal homeostasis during HFD [4, 5], elevation of IL-17 is also associated with obesity-related rheumatoid arthritis, multiple sclerosis, psoriasis, and cancer [53], suggesting that a delicate IL-17 site-specific balance is required to achieve homeostasis. Here, we demonstrated that indigo therapy resulted in a reduction of intestinal IL-17 producing Th17 cells. Previous work in human T helper cells has shown that AhR-ligation not only decreased the number of Th17 cells, but also primed naive CD4+ T cells toward IL-22 production [54]. Similarly, the loss of AhR in mice results in an increased number of small intestine Th17 cells, which may be related to the role of AhR in suppressing microbiota-mediated Th17 cell differentiation, specifically by segmented filamentous bacteria [55, 56]. AhR can also inhibit the skew toward Th1 and Th17 responses by altering the activation and cytokine production of dendritic cells [57]. In the future, it will be interesting to determine how indigo impacts T-cell metabolic reprogramming which might also explain its effects on Th1 and Th17 differentiation [58].

An increasing number of studies have elucidated means by which immunological changes in the intestine can contribute to VAT inflammation and overall worsened metabolic disease [59]. In addition to the observed decrease in inflammatory changes in the intestine in HFD-fed mice, we investigated whether this treatment influences immune changes in the VAT. Specifically, AhR signaling in macrophages has been reported to promote anti-inflammatory M2 phenotype with increased IL-10 production in mouse models of lupus, associated with reduced production of anti-dsDNA autoantibodies [28]. Consistently, we observed that indigo can directly increase IL-10 production and M2 polarization by macrophages in VAT possibly via AhR activation. It will be interesting to assess whether indigo as an AhR agonist has a capacity to alter immunity against nucleic acid targeting pathways in IR or whether it also has effects on lupus [33]. Similar to the previously observed protective role for AhR against hepatic steatosis and hepatic inflammation in HFD mice [50, 60], indigo supplementation decreased liver triglyceride content and hepatic inflammation by increasing IL-10 and M2-like macrophages. Indigo feeding also decreased serum triglyceride levels which is consistent with the previous report that the AhR acts as an inhibitor of triglyceride synthesis [61]. Our data revealed that indigo could decrease recruited hepatic macrophages which were previously reported to be increased in numbers by sixfold in obese mice [62] and potentially reduce liver damage during DIO. Overall, indigo feeding in HFD mice effectively protected against IR and obesity-related inflammation in metabolic tissues including the gut, VAT, and liver.

Since HFD-related gut barrier dysfunction is a source of gut, VAT and liver inflammation and IR [3, 9] and AhR may have beneficial effects on the intactness of the gut epithelial barrier mediated by IL-22 [20], we investigated similar immune-mediated mechanisms for indigo in glucose homeostasis. In this study, we confirmed that indigo activates the classical AhR target gene *Cyp1a1* and mediates the restoration of gut barrier function by increasing IL-22 production in the small intestine. HFD-Indigo fed mice displayed increased IL-22 secretion in CD4+ T cells. IL-22 has been previously shown to be reduced in the intestine of obese mice and restoration of IL-22 decreases metabolic abnormality by targeting intestinal permeability and endotoxemia [25, 63]. Improvement in gut barrier function and increased IL-22 levels was associated with increases in tight junction gene expression, including *claudin 1* and *ZO1*, and upregulated expression of antimicrobial peptide *Reg3g* and mucus secretion gene *Muc2*, all of which are known to be downregulated during obesity [4, 26]. In addition to IL-22, we identified that indigo also increases IL-10 in the intestine. Previous studies have shown that AhR regulates IL-10 receptor expression on intestinal epithelia by regulatory type 1 and natural killer cells and is important for intestinal Treg homing and anti-inflammatory function [64–67].

Next, we investigated the cellular source of IL-22 induced by indigo in the small bowel. AhR is directly involved in IL-22 production by both ILC3s and IL-22 producing Th17/Th22 cells [68, 69]. During HFD, reduction in IL-22 is attributed to decreased amounts of IL-22 from NKp46+ CD4− ILC3s and Th17/Th22 cells [3, 70]. Here, we showed that indigo can induce IL-22 production from differentiated gut lymphoid system related CD4+ T cells in vitro and small intestine explants of HFD-fed mice. Interestingly, indigo seemed to be acting preferentially on T cells in the intestine instead of ILC3s as no significant beneficial effects on glucose homeostasis were observed in HFD-Indigo fed Rag1^null^ mice, which are deficient in T and B cells while retaining ILCs [3]. Accordingly, unlike wild-type mice, no differences were observed in IL-22 production from small bowel tissue explants of Rag1^null^ mice. Thus, while we cannot completely exclude a role for ILC3 responses, we concluded that the effects of indigo in glucose homeostasis have an important dependence on the adaptive immune system, specifically by IL-22 producing T cells. The result is compatible with previous reports, as AhR-deficiency in mice resulted in reduced IL-22-secreting CD4+ T cells [56] and more specifically, a psoriasis model demonstrated the requirement of AhR for IL-22 production by Th17, but not by ILC3 and γδ+ T cells [71]. Indigo-induced alterations to the gut microbiota toward an increased proportion of Lactobacillus bacteria may also contribute to the increase in IL-22 production in HFD-Indigo fed mice. Due to indigo’s structural similarity to other indole-based compounds like tryptophan and its metabolites, it may potentially influence Lactobacillus-mediated metabolism to generate indole derivatives that activate the AhR and downstream IL-22 production [44].

Overall, our work shows that indigo is a novel orally ingested plant-derived agent that is able to alleviate HFD-induced gut barrier dysfunction and low-grade inflammation in the gut, VAT, and liver. Improvement in gut barrier function leads to reduced inflammation in metabolic tissues and overall amelioration of IR. The overall beneficial effects of indigo on metabolic homeostasis are dependent on IL-22 secretion by the adaptive immune system, as well as direct and indirect effects on IL-10 production on immune cells residing inside the intestine, liver and fat. Interestingly, while AhR whole-body deficiency has been shown to protect mice from diet-induced adiposity [48], due to the multifaceted role of AhR, AhR activation via indigo within the small intestine specifically prevents systemic inflammation and subsequent metabolic tissue inflammation and IR. One potential caveat with our study is the use of a more extreme high-fat content (60 kcal% fat) in the diet compared to a more traditional Western diet (45 kcal% fat) consumed by humans. While many studies have used this very HFD diet to investigate the role of the immune system, specifically within the intestine, in obesity and IR [35, 72–74], future investigation is warranted to investigate whether indigo has similar anti-inflammatory effects, or gut microbial effects in tissues during lower fat intake such as a 35–45 kcal% fat diets. Moreover, more studies are needed to better contrast gut microbial impact of a 60 kcal% HFD compared to a 45 kcal% HFD diet [75]. Furthermore, additional preclinical and clinical studies are needed to validate the effects and safety of indigo and related compounds in humans. While Indigo alone is commonly used as a coloring agent and is thought to possess low acute and chronic toxicity, one report noted mild-liver dysfunction and a single case of pulmonary arterial hypertension in ulcerative colitis patients treated with its parent compound *Indigo Naturalis* [30, 76]. Nonetheless, our study provides preclinical evidence that naturally occurring AhR ligands have the capacity to target multiple important contributing facets linked to IR, including gut barrier function and metabolic tissue inflammation. Indigo and other natural AhR ligands with immunomodulatory effects may represent a novel class of therapeutics for the control of obesity-related complications, including IR.

## Supplementary information

Lin and Luck et al. Indigo Supplementary Information Revised
