# Supplementary material for: Aryl hydrocarbon receptor agonist indigo protects against obesity-related insulin resistance through modulation of intestinal and metabolic tissue immunity
Source: Int J Obes (Lond). 2019 Apr 3;43(12):2407–21. doi: 10.1038/s41366-019-0340-1 (PMC6892742; doi:10.1038/s41366-019-0340-1)
Supplement: Supplementary file 1 — Lin and Luck et al. Indigo Supplementary Information Revised [file 41366_2019_340_MOESM1_ESM.pdf]

Supplementary Information

Aryl Hydrocarbon Receptor Agonist Indigo Protects Against Obesity-Related Insulin Resistance through Modulation of Intestinal and Metabolic Tissue Immunity

Supplementary Methods

Study Design

We utilized diet-induced obesity (DIO) mouse models to examine for changes in insulin resistance with indigo intervention. In our mouse model of DIO, wild-type (WT) mice were randomly assigned to receive either HFD (Research Diets, 60% kcal fat) or HFD-Indigo (Research Diets Inc., 300mg/kg/day) starting at 6 weeks of age for up to 16 weeks. Primary outcomes of metabolic assessments in mouse studies included glucose and insulin tolerance. Primary immune analysis of mouse studies included the immunophenotyping of T cell, innate lymphoid cell, macrophage populations, and their production of pro-inflammatory cytokines by flow cytometry and expression of barrier function related genes. Investigators were not blinded for glucose and metabolic testing or flow cytometry analysis. Histological specimens and microscopy were analyzed by a blinded pathologist. The sample size and replicates of experiments are indicated in the figure legends. Sample size was not predetermined, but we performed experiments with group sizes based on literature documentation of similar experiments. An extreme studentized deviate method (Grubbs' test) was performed with GraphPad software to assess for statistical outliers.

Plant-Derived Compounds Preparation

A voucher specimen (CGU-SB-1 and CGU-ES-1) were deposited in the herbarium of Chang

Gung University, Taoyuan, Taiwan. *Scutellaria baicalensis* (3 Kg) was extracted with EtOH (6 L × 3, 70°C) and concentrated to give brown syrup (625 g). The syrup was suspended in H_2O and partitioned with EtOAc and n-BuOH, successively. Baicalein (4149 mg) and wogonin (1129 mg) were purified from the EtOAc extract (64 g) by silica gel column chromatography. n-BuOH extract (91 g) was applied on Dianion HP-20 column to get baicalin (3005 mg) and wogonoside (770 mg), separately. *Ephedra sinica* (600 g) was extracted with EtOH (5 L × 3, 70°C) and concentrated to give dark brown syrup (59 g).

Metabolic Cage Studies

We placed mice in automated metabolic cages (Oxymax Systems, Columbus Instruments) for 48 h with airflow held constant at 0.5 L/min and monitored for food and water intake after 14 weeks HFD or HFD-Indigo. We measured metabolic activity using indirect calorimetry, recording maximal O2 consumption (VO2), CO2 production (VCO2) normalized to body weight. Respiratory exchange ratio (RER) was derived from VCO2/VO2. Energy expenditure (kcal) was calculated as calorific value (CV) xVO2. CV is 3.815 + 1.232 x RER. Food and water intake are calculated for light and dark measurements as an average over 24 hours by combining light and dark measurements. Ambulatory activity was measured by the breaking of infrared laser beams in the XYZ axis.

Histology

We enumerated CLS in the VAT by counting the number of adipocytes surrounded by immune cells identified on H&E staining per 100X low power field. We used the straight-line tool in the Leica Application Suite software to measure fat cell diameter. Analysis of histochemical stains

was performed in a blinded fashion by a certified pathologist.

Isolation of Stromal Vascular Cells from Visceral Adipose Tissue

Epididymal VAT pads were dissected, mashed and digested in collagenase (0.2 mg/ml, DMEM/60min/37°C, manual shaking every15min, Sigma). Cells were washed, pelleted, filtered through a 70 µm filter and then followed by red blood cell lysis and washed to obtain SVC.

Isolation of Bowel-Associated Immune Cells

For isolation of small intestine lamina propria immune cells, we used the protocol previously described (1) and processed 10 cm from the distal end of the small bowel (jejunum and ileum). Small and large intestines were extracted, removing mesentery fat and Peyer's patches, and the intestine was cut open longitudinally and into pieces of around 5 mm. Tissue pieces were washed twice with gentle vortexing for a few seconds in ice-cold buffer (HBSS (Gibco) supplemented with 2% FBS (PAA) and 15 mM HEPES pH 7.4). Bowel pieces to were transferred to an EDTA-containing buffer (HBSS (Gibco) supplemented with 10% FBS (PAA), 5 mM EDTA, 15 mM HEPES, buffered with NaOH at pH 7.4) and shaken vigorously at 37˚C for 20 minutes, vortexed gently for a few seconds. This wash step was repeated two times. Gut pieces were then washed three times in cold HBBS buffer (Gibco) to remove residual EDTA before transfer into RPMI 1640 supplemented with 10% FBS (PAA), 15 mM HEPES pH7.4, Collagenase type IV (5 mg/ml, Sigma) and DNase I (0.5 mg/ml, Roche) for digestion of tissue for 1 hour at 37 °C with shaking. The resulting suspension of lamina propria immune cells collected from the previous step were filtered through a 100 and 70 µm nylon cell strainer to obtain a single cell suspension. Finally, cells were washed and resuspended in ice-cold FACS buffer containing PBS

supplemented with 2% FBS and analyzed by flow cytometry.

In vitro IL-22-producing CD4+ T cells Differentiation

We harvested mesenteric lymph nodes from mice and mashed lymph nodes through a sterile 70-μm nylon cell strainer to achieve single-cell suspensions in RPMI 1640 containing 10% FCS. Cells were resuspended and purified for naïve CD4 T cells (Stemcell Technologies) and cultured under 0.5 μg/ml anti-mouse CD3ε (BioLegend), 5 μg/mL anti-mouse CD28 (BioLegend), 50 ng/mL IL-6 (BioLegend), 1ng/mL TGF-β1 (BioLegend), 10 μg/mL anti-mouse IL-4 (BioLegend), 10 μg/mL anti-mouse IFN-γ (BioLegend), and 5 ng/ml mouse IL-23 (BioLegend), with 0.05% DMSO, 50μM indigo, or 300nM FICZ for 4 days. Supernatants were collected to assess IL-22 levels by ELISA (BioLegend).

Flow Cytometry

We stained single-cell suspensions for 30 min on ice with commercial antibodies. We gated ILCs as described (2). Flow cytometry antibodies including CD45.2 (104), CD3 (17A2), CD4 (GK1.5), CD8 (53-6.7), CD8b (YTS156.7.7), γδTCR (GL3), Foxp3 (150D), IL-17 (TC11-18H10.1), IFNγ (XMG1.2), IL-22 (Poly5164), CD11b (M1/70), F4/80(BM8), CD11c (N418), CD206 (C068C2), CD80 (16-10A1), NKp46 (CD335, 29A1.4), GATA3 (16E10A2), Lineage (145-2C11, RB6-8C5, M1/70, RA3-6B2, Ter-119) and CD127 (IL-7Rα, A7R34) were purchased from Biolegend. RORγt (AFKJS-9) was purchased from eBioscience. Intracellular staining was performed using a Foxp3 staining buffer kit (eBioscience). We acquired data from the Fortessa flow cytometer (BD Biosciences) and analyzed the data with FlowJo software (Tree Star).

RNA Isolation and Quantitative Real Time-PCR

We extracted total RNA from the small intestine, liver using the RNeasy Mini Kit (QIAGEN), and for VAT and BAT, we used the RNeasy Lipid Extraction kit (QIAGEN). We reverse-transcribed the RNA by SensiFAST cDNA synthesis kit (Bioline). We performed qPCR with a QuantStudio 7 Flex Real-Time PCR System (Applied Biosystems) using SYBR Green Master Mix reagent (Applied Biosystems). Each sample was checked in triplicate and normalized to housekeeping genes, *Actb*. We calculated relative fold changes in gene expression normalized to *Actb* by the DDCT method using the equation $2^{-\triangle\triangle CT}$. The results are shown as fold changes compared to the control group.

Gut Permeability Assays

Mice were fasted overnight and then orally gavaged with FD4 (0.4 mg/g body weight (BW) of a 100 mg/mL solution; Sigma-Aldrich). After 4 hours, 120 µL of leg vein blood was collected, and plasma fluorescence was measured at the excitation wavelengths of 485 nm and the emission wavelengths of 528 nm, compared with a standard curve using FD4 in normal plasma (3).

16S rRNA gene sequencing

The V4 hypervariable region of the 16S rRNA gene is amplified using a universal forward sequencing primer and a uniquely barcoded reverse sequencing primer to allow for multiplexing (4). Amplification reactions are performed using 12.5 uL of KAPA2G Robust HotStart ReadyMix (KAPA Biosystems), 1.5 uL of 10 uM forward and reverse primers, 7.5 uL of sterile water and 2 uL of DNA. The V4 region was amplified by cycling the reaction at 95°C for 3 minutes, 22x cycles of 95°C for 15 seconds, 50°C for 15 seconds and 72°C for 15 seconds,

followed by a 5 minute 72°C extension. All amplification reactions were done in triplicate, checked on a 1% agarose TBE gel, and then pooled to reduce amplification bias. Pooled triplicates were quantified using PicoGreen and combined by even concentrations. The library was then purified using Ampure XP beads and loaded on to the Illumina MiSeq for sequencing, according to manufacturer instructions (Illumina, San Diego, CA). Sequencing is performed using the V2 (150bp x 2) chemistry. A single-species (*Pseudomonas aeruginosa* DNA), a mock community (Zymo Microbial Standard: https://www.zymoresearch.de/zymobiomics-community-standard) and a template-free negative control were included in your sequencing run.

Analysis of the bacterial microbiome

The UNOISE pipeline, available through USEARCH USEARCH v10.0.240 and vsearch v2.5.0, was used for sequence analysis (5-7). The last base was removed from all sequences. Sequences were assembled and quality trimmed using –fastq_mergepairs and –fastq_filter, with a –fastq_maxee set at 1.0. Sequences shorter than 233 base pairs were discarded. The trimmed data was then processed following the UNOISE pipeline. Sequences were first de-replicated and sorted to remove singletons, then denoised and chimeras were removed using the unoise3 command. Assembled sequences were mapped back to the chimera-free denoised sequences at 97% identity OTUs. Taxonomy assignment was executed using SINTAX, available through USEARCH, and the UNOISE compatible Ribosomal Database Project (RDP) database version 16, with a minimum confidence cutoff of 0.8 (7). OTU sequences were aligned using PyNast accessed through QIIME (8). Sequences that did not align were removed from the dataset and a phylogenetic tree of the filtered aligned sequence data was made using FastTree (9). The bar chart was generated in R version 3.4.4 (10) using the packages funrar version 1.2.2 (11), dplyr

version 0.7.6 (12), reshape2 version 1.4.3 (13), and ggplot2 version 2.3.0 (14). STAMP version 2.1.5 (15) was used to generate the box plots and the PCA plot and group mean comparison was performed using the White's non-parametric *t*-test (16).

References

1. Fritz JH, Rojas OL, Simard N, McCarthy DD, Hapfelmeier S, Rubino S, et al. Acquisition of a multifunctional IgA+ plasma cell phenotype in the gut. Nature. 2011;481(7380):199-203.

2. Mizoguchi A. Healing of intestinal inflammation by IL-22. Inflammatory bowel diseases. 2012;18(9):1777-84.

3. Dong CX, Zhao W, Solomon C, Rowland KJ, Ackerley C, Robine S, et al. The intestinal epithelial insulin-like growth factor-1 receptor links glucagon-like peptide-2 action to gut barrier function. Endocrinology. 2014;155(2):370-9.

4. Caporaso JG, Lauber CL, Walters WA, Berg-Lyons D, Huntley J, Fierer N, et al. Ultra-high-throughput microbial community analysis on the Illumina HiSeq and MiSeq platforms. ISME J. 2012;6(8):1621-4.

5. Edgar RC. Search and clustering orders of magnitude faster than BLAST. Bioinformatics. 2010;26(19):2460-1.

6. Edgar RC. UPARSE: highly accurate OTU sequences from microbial amplicon reads. Nat Methods. 2013;10(10):996-8.

7. Wang Q, Garrity GM, Tiedje JM, Cole JR. Naive Bayesian classifier for rapid assignment of rRNA sequences into the new bacterial taxonomy. Appl Environ Microbiol. 2007;73(16):5261-7.

8. Caporaso JG, Kuczynski J, Stombaugh J, Bittinger K, Bushman FD, Costello EK, et al. QIIME allows analysis of high-throughput community sequencing data. Nat Methods. 2010;7(5):335-6.

9. Price MN, Dehal PS, Arkin AP. FastTree: computing large minimum evolution trees with profiles instead of a distance matrix. Mol Biol Evol. 2009;26(7):1641-50.

10. Team RC. R: A language and environment for statistical computing 2018 [Available from: https://www.R-project.org/.

11. Violle C, Thuiller W, Mouquet N, Munoz F, Kraft NJB, Cadotte MW, et al. Functional Rarity: The Ecology of Outliers. Trends Ecol Evol. 2017;32(5):356-67.

12. Hadley Wickham RF, Lionel Henry and Kirill Müller. dplyr: A Grammar of Data Manipulation 2018 [Available from: https://CRAN.R-project.org/package=dplyr.

13. H W. Reshaping data with the reshape package. Journal of Statistical Software 2007;21(12):1-20.

14. H W. ggplot2: elegant graphics for data analysis. Springer. 2016.

15. Parks DH, Tyson GW, Hugenholtz P, Beiko RG. STAMP: statistical analysis of taxonomic and functional profiles. Bioinformatics. 2014;30(21):3123-4.

16. White JR, Nagarajan N, Pop M. Statistical methods for detecting differentially abundant features in clinical metagenomic samples. PLoS Comput Biol. 2009;5(4):e1000352.

Supplementary Table and Figures

Table S1. Primer sets used for RT-PCR

Gene	Forward Primer Sequences	Reverse Primer Sequences
Actb	GCCCTGAGGCTCTTTTCCAG	TGCCACAGGATTCCATACCC
Ucp1	GGCAAAAACAGAAGGATTGC	TAAGCCGGCTGAGATCTTGT
Prdm16	GAAGTCACAGGAGGACACGG	CTCGCTCCTCAACACACCTC
Pgc-1	AATGCAGCGGTCTTAGCACT	TTTCTGTGGGTTTGGTGTGA
Tnfa	TACTGAACTTCGGGGTGATTGGTCC	CAGCCTTGTCCCTTGAAGAGAACC
Il10	TGAGGCGCTGTCGTCATCGATTTCTCCC	ACCTGCTCCACTGCCTTGCT
Il22	GTGGGATCCCTGATGGCTGTCCTGCAG	AGCGAATTCTCGCTCAGACTGCAAGCAT
Cyp1a1	CCTCATGTACCTGGTAACCA	AAGGATGAATGCCGGAAGGT
Il6	CCGGAGAGGAGACTTCACAG	GGAAATTGGGGTAGGAAGGA
Reg3g	TTCCTGTCCTCCATGATCAAA	CATCCACCTCTGTTGGGTTC
ZO1	ACCCGAAACTGATGCTGTGGATAG	AAATGGCCGGGCAGAACTTGTGTA
claudin 1	CTGGGTTTCATCCTGGCTTC	TTGATGGGGGTCAAGGGGT
Ahr	GGCTTTCAGCAGTCTGATGTC	CATGAAAGAAGCGTTCTCTGG
Arg1	CTCCAAGCCAAAGTCCTTAGAG	AGGAGCTGTCATTAGGGACATC
Nos2	GTTCTCAGCCCAACAATACAAGA	GTGGACGGGTCGATGTCAC
Muc2	GCCCGTGGAGTCGTACGTGC	TTGGGGCAGAGTGAGGCGGT

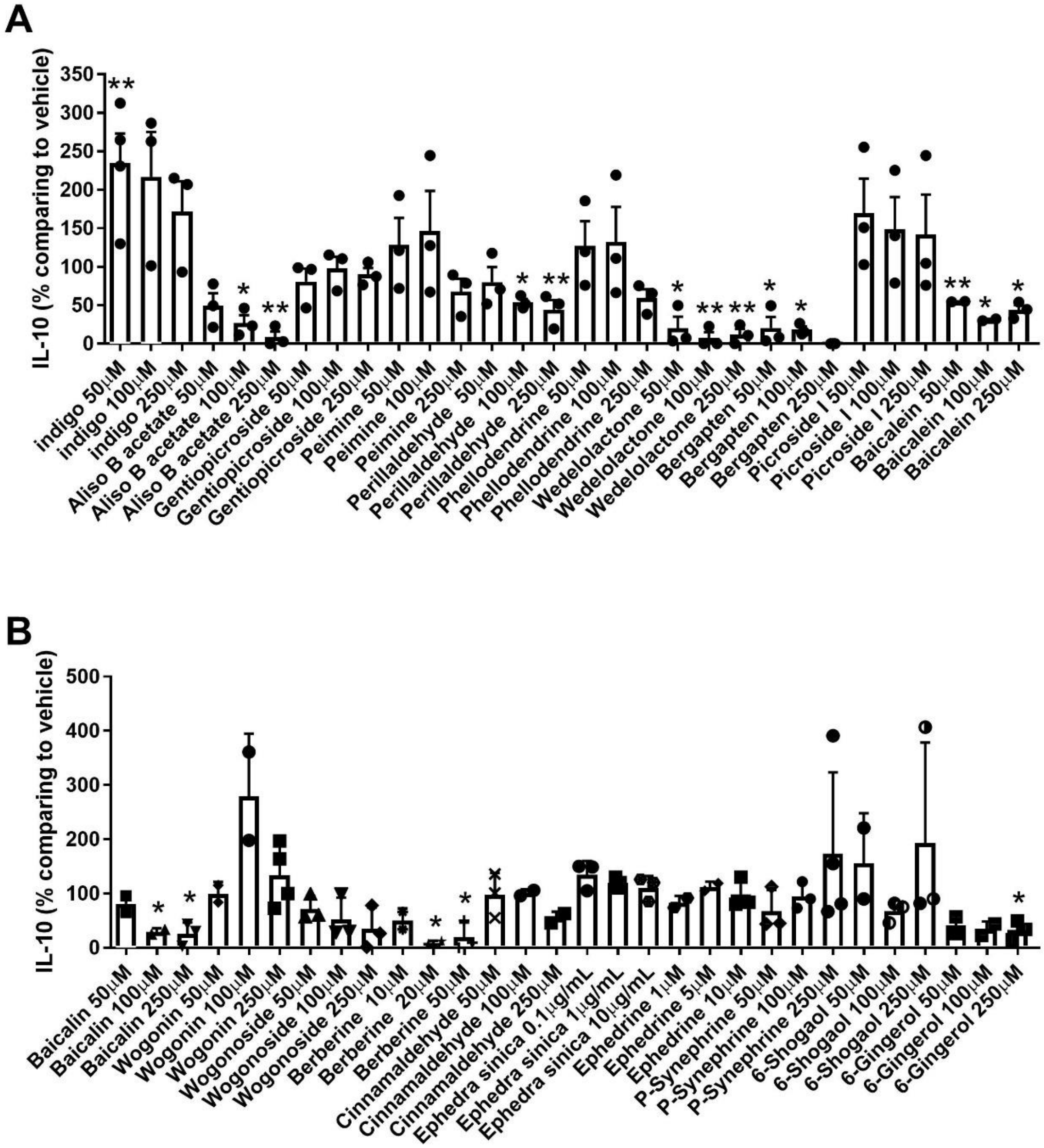

Figure S1. Chemical screen of plant derivatives production of anti-inflammatory cytokine IL-10 in HFD-induced immune cells in VAT.

IL-10 production in the VAT SVC of HFD mice treated with drug candidates or their vehicle (DMSO, EtOH, or water) for 3 days (n=2-4 experiments pooled from 15 mice per experiment). We tested three doses of each drug. Drug concentrations: Indigo, aliso B acetate, gentiopicroside, peimine, perillaldehyde, phellodentrine, wedelolactone, bergapten, picroside I, baicalein,

baicalin, wogonin, wogonoside, cinnamaldehyde, p-synephrine, 6-shogaol and 6-gingerol are 50, 100 and 250μM; berberine is in 10, 20 and 50μM; *Ephedra sinica* is in 0.1,1 and 10μg/mL; Ephedrine is in 1, 5, and 10μM. Data in bar graphs are presented as mean ± SEM. $*p < 0.05$, $**p<0.01$, $*p=0.0286$ Mann-Whitney test for wedelolactone 100 μM.

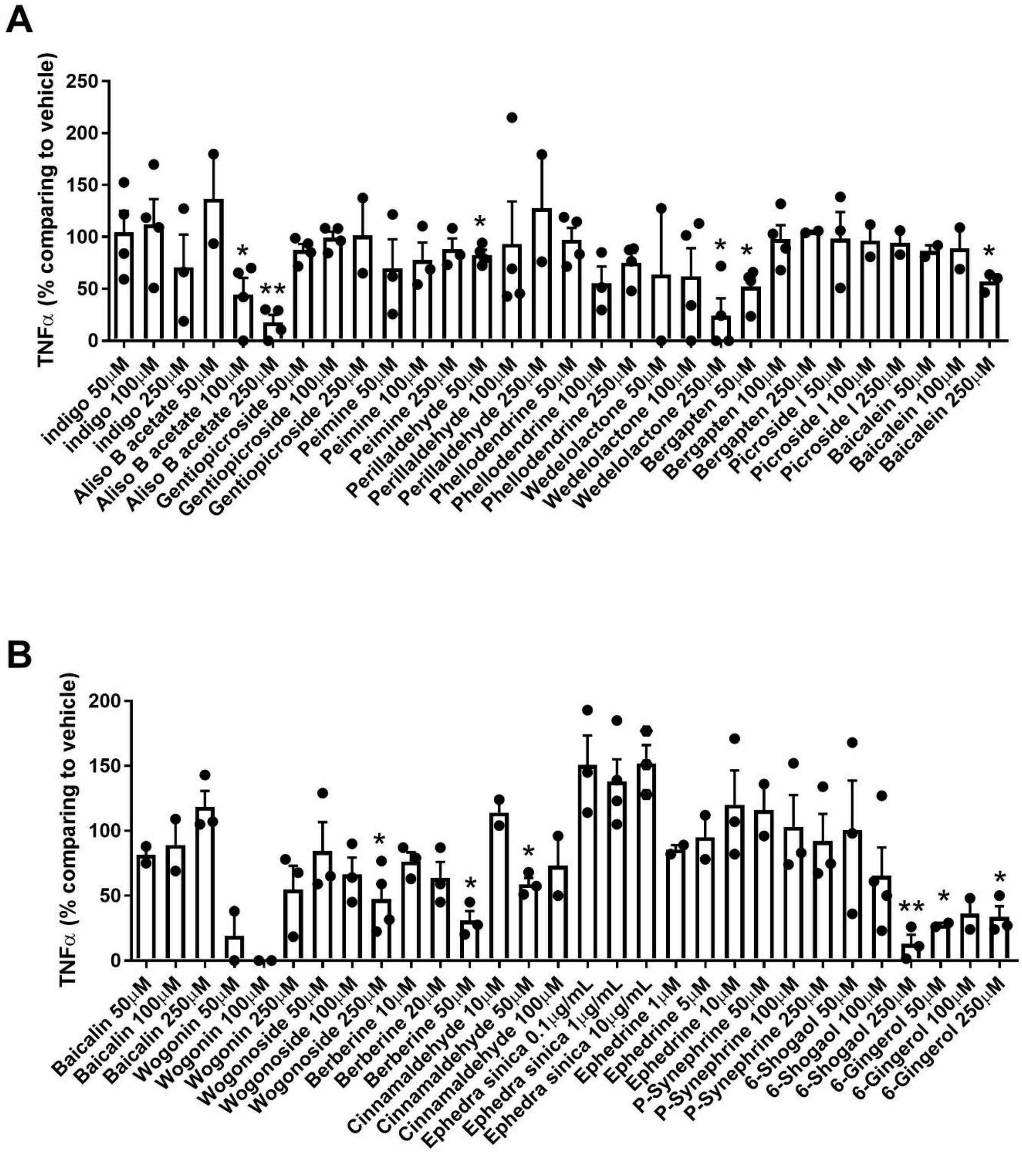

Figure S2. Chemical screen of plant derivatives identifies compounds that could decrease TNFα in HFD-induced immune cells in VAT.

TNFα production in the VAT SVC of HFD mice treated with drug candidates or their vehicle (DMSO, EtOH, or water) for 3 days (n=2-4 experiments pooled from 15 mice per experiment).

We tested three doses of each drug. Drug concentrations: Indigo, aliso B acetate, gentiopicroside, peimine, perillaldehyde, phellodentrine, wedelolactone, bergapten, picroside I, baicalein, baicalin, wogonin, wogonoside, cinnamaldehyde, p-synephrine, 6-shogaol and 6-gingerol are 50, 100 and 250μM; berberine is in 10, 20 and 50μM; *Ephedra sinica* is in 0.1,1 and 10μg/mL; Ephedrine is in 1, 5, and 10μM. Data in bar graphs are presented as mean ± SEM. *p < 0.05, **p<0.01.

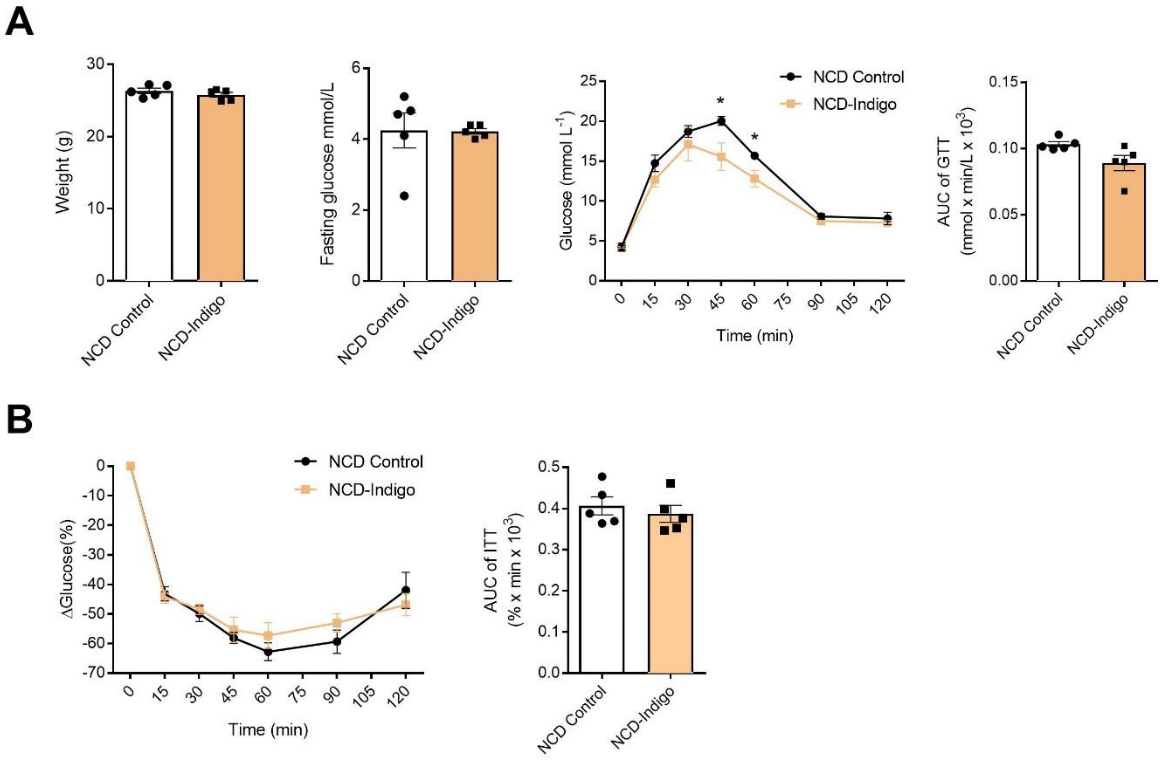

Figure S3. Metabolic testing of mice fed an Indigo supplemented normal chow diet (NCD-Indigo). A) Weight (far left), fasting glucose (middle left), glucose tolerance test (GTT) (middle right), area under curve for GTT (far right) of mice fed NCD-Indigo compared to chow controls. B) Insulin tolerance test (ITT) and area under the curve for NCD-Indigo fed mice compared to NCD controls. Data in bar graphs are presented as mean ± SEM. n=5/group.

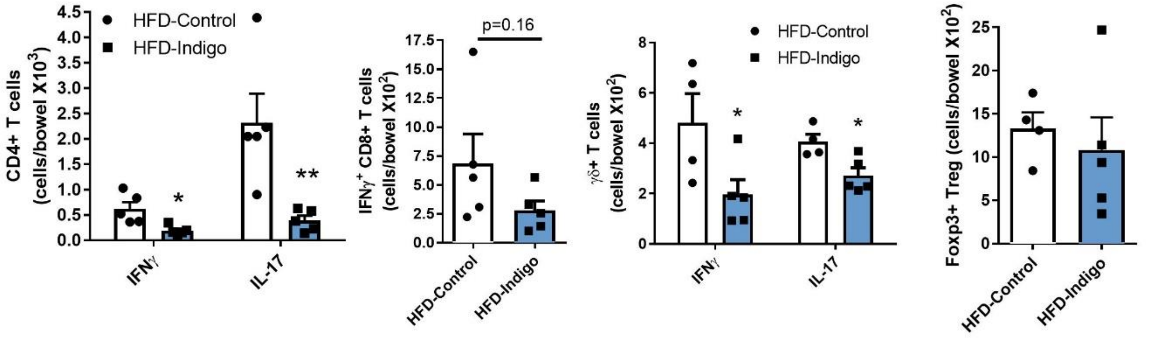

Figure S4. Absolute numbers of intracellular staining of cytokines and Foxp3 transcription factor in the small intestine lamina propria T cell populations of wildtype mice after 14 weeks of HFD or HFD-Indigo. Data in bar graphs are presented as mean ± SEM. n=5/group. $*p < 0.05$, $**p<0.01$.

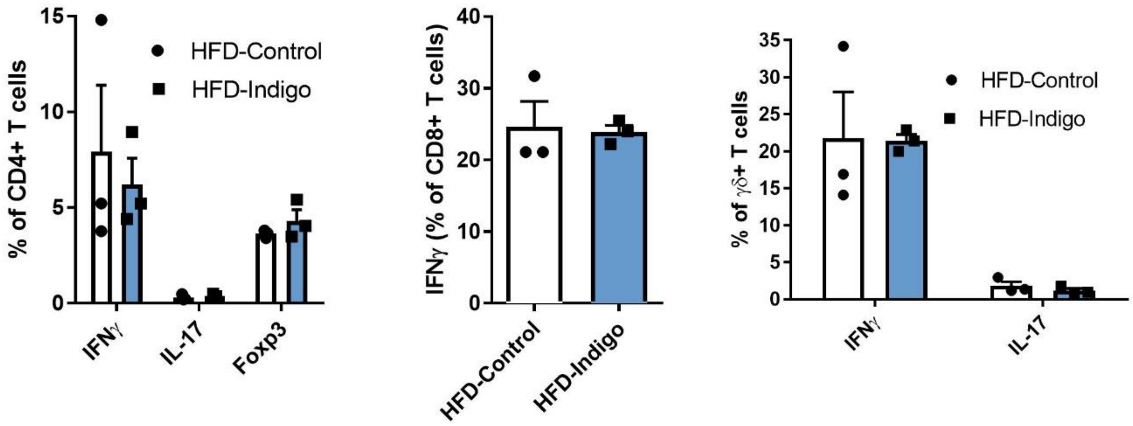

Figure S5. Percentages of splenic immune cells in HFD-Control and HFD-Indigo WT mice, including CD4+ T cell subsets including Foxp3+ Tregs (left), IFNγ-producing CD8+ T cells (middle) and γδ+ T cell subsets (right). Data in bar graphs are presented as mean ± SEM. n=3/group.

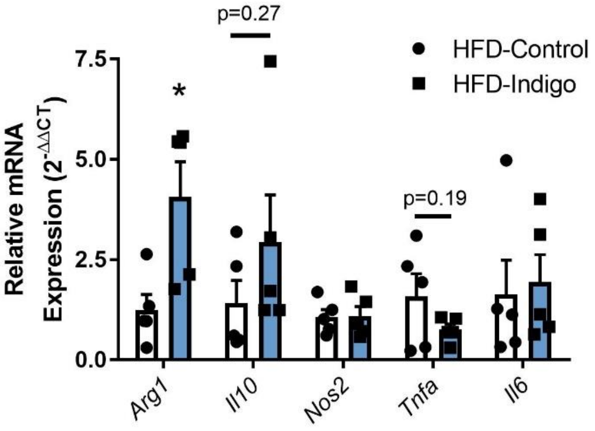

Figure S6. Indigo Promotes M2-like Macrophage Marker *Arg1* Expression during HFD in the small intestine.

mRNA expression of M2-like macrophage marker *Arg1, Il10, iNOS, Tnfa* and *Il6* in the small intestine whole tissue after HFD-Control and HFD-Indigo treatment for 14 weeks (n=5/group, *p=0.032 Mann-Whitney test). Data in bar graphs represent mean ± SEM.

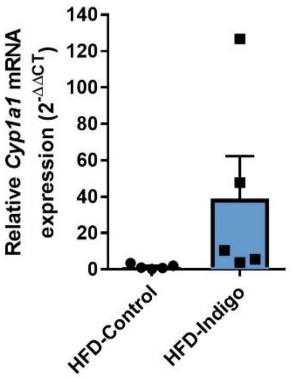

Figure S7. *Cyp1a1* mRNA expression of VAT whole tissue from HFD-Control and HFD-Indigo mice. (n=5/group). Data in bar graphs represent mean ± SEM.

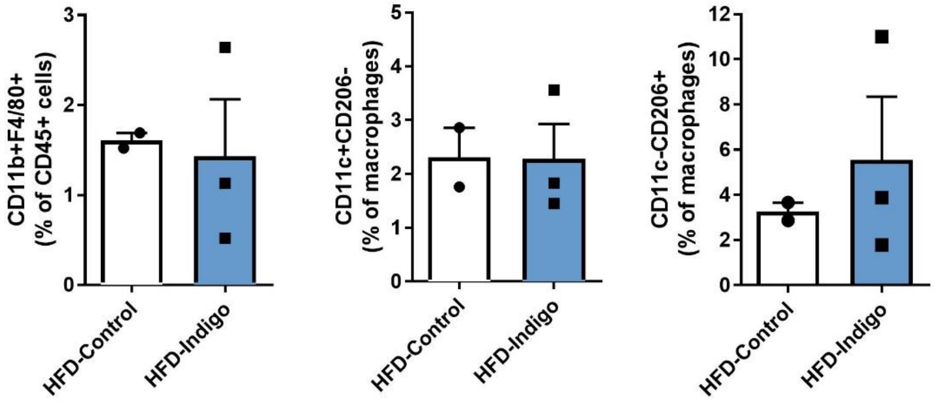

Figure S8. Percentages of splenic immune cells in HFD-Control and HFD-Indigo WT mice, including CD11b+F4/80+ cells (left), CD11c+CD206- cells (middle), CD11c-CD206+ cells (right). Data in bar graphs are presented as mean ± SEM. (n=2-3/group)

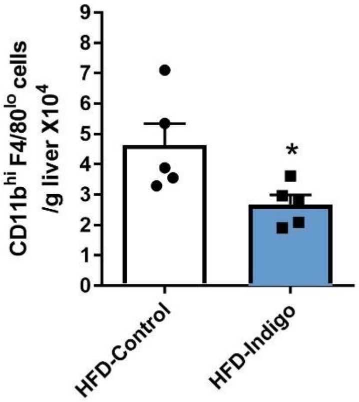

Figure S9. Flow cytometry analysis of absolute numbers of CD11b^{hi} F4/80^{lo} recruited hepatic macrophages in liver of mice after 16 weeks of HFD or HFD-Indigo. Data in bar graphs are presented as mean ± SEM. (n=5/group). *$p < 0.05$.

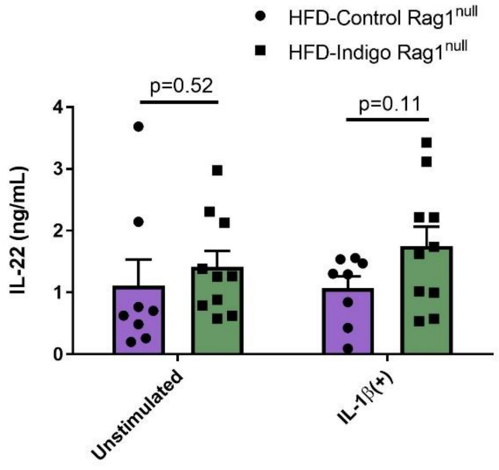

Figure S10. IL-22 in small bowel tissue explant with or without IL-1β stimulation in HFD-Control and HFD-Indigo Rag1^{null} mice. Data in bar graphs are presented as mean ± SEM. (n=8 in HFD-Control, n=10 in HFD-Indigo Rag1^{null} mice).

A

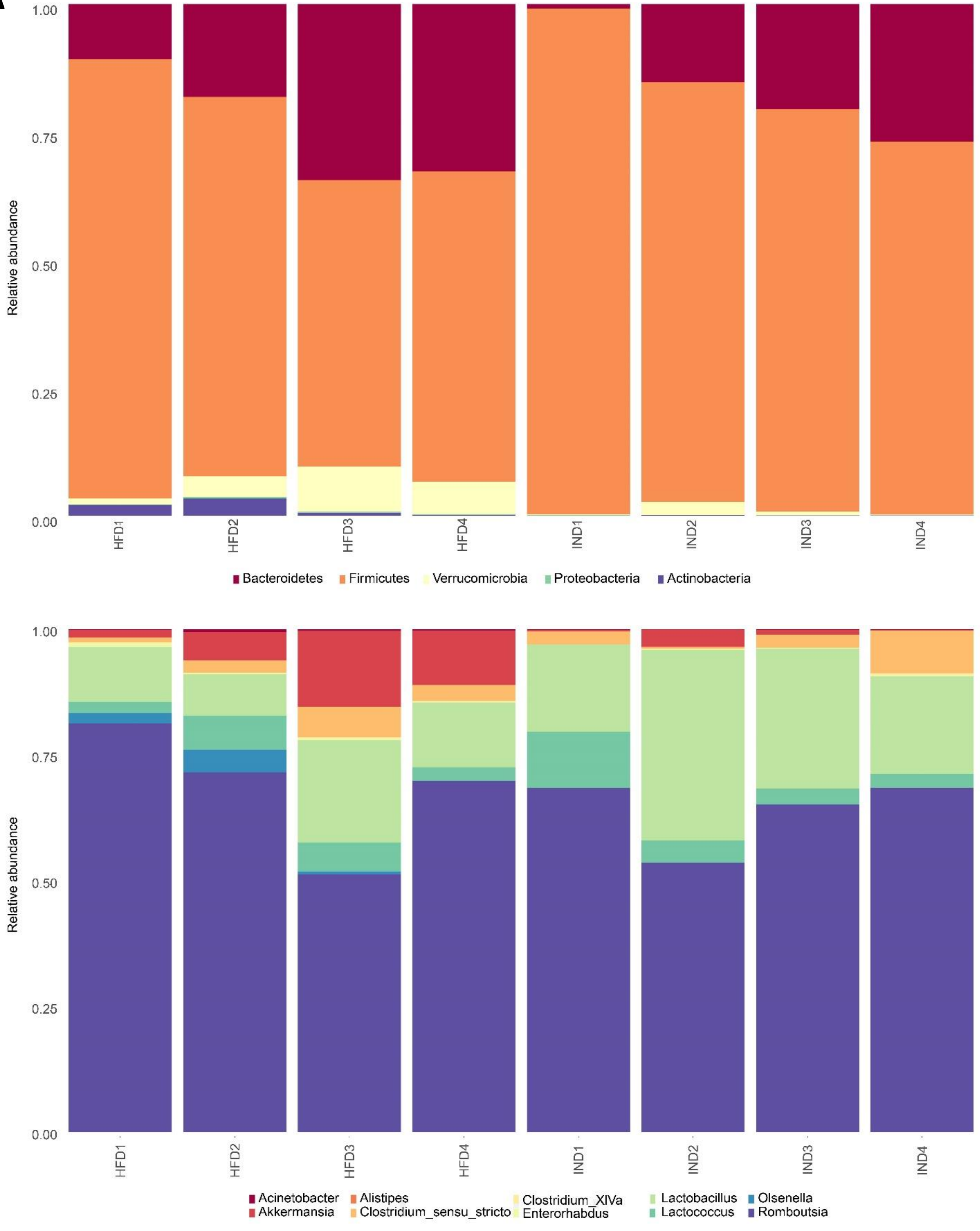

1.00
0.75
0.50
0.25
0.00
Relative abundance
HFD1
HFD2
HFD3
HFD4
IND1
IND2
IND3
IND4
Bacteroidetes
Firmicutes
Verrucomicrobia
Proteobacteria
Actinobacteria
1.00
0.75
0.50
0.25
0.00
Relative abundance
HFD1
HFD2
HFD3
HFD4
IND1
IND2
IND3
IND4
Acinetobacter
Akkermansia
Alistipes
Clostridium_sensu_stricto
Clostridium_XIVa
Enterorhabdus
Lactobacillus
Lactococcus
Olsenella
Romboutsia

B

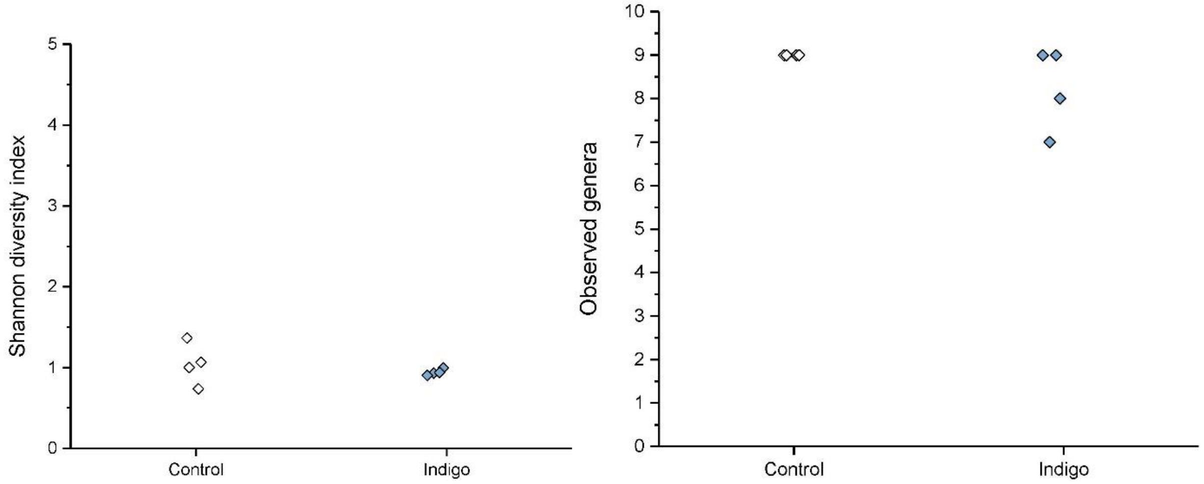

C

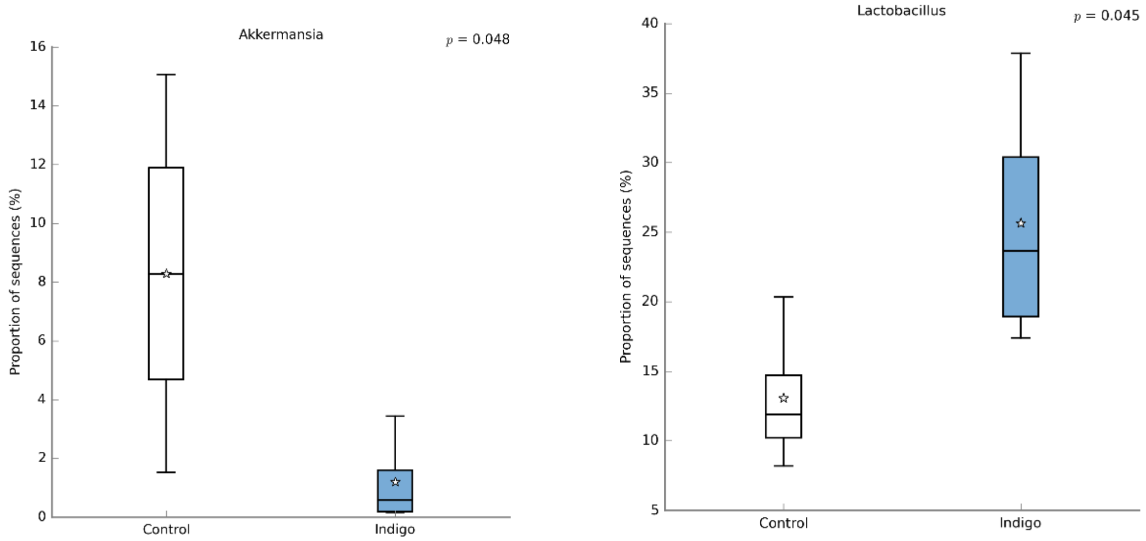

Figure S11. Small bowel microbiome profile of HFD-Indigo fed mice compared to HFD-fed Controls. A) Bar charts at the phylum and genus levels, **B)** Shannon diversity index and observed genera analyses, and **C)** Proportions of significant genera, Akkermansia and Lactobacillus, in HFD-Indigo fed mice compared to HFD-fed controls. (n=4 mice/group, *$p<0.05$ White's non-parametric *t*-test).
